# Family support during childhood as a predictor of mate retention and kin care in adults during the COVID-19 pandemic in Brazil: an exploratory study

**DOI:** 10.3389/fpsyg.2023.1276267

**Published:** 2023-12-19

**Authors:** Natália de Araújo Miranda Tasso, Felipe Nalon Castro

**Affiliations:** Department of Physiology and Behavior, Federal University of Rio Grande do Norte, Natal, Brazil

**Keywords:** harshness, unpredictability, pandemic, fundamental social motives, life-history theory

## Abstract

**Introduction:**

Experiences during development help to explain behavior expression in adulthood.

**Aims:**

In this study, we explored how unpredictability and harshness experienced during childhood may have impacted the occurrence of reproductive milestones in adulthood and the expression of fundamental motives related to self-protection, disease avoidance, mate seeking, mate retention, and kin care (children/family) during the pandemic.

**Methods:**

This was an exploratory study with 438 participants. Through the administration of online questionnaires, participants were assessed and categorized based on their childhood experiences, resulting in three groups: low unpredictability, high unpredictability with family support, and high unpredictability without family support.

**Results:**

We found that family support experienced during childhood predicts a slow life-history strategy. This involves an emphasis on growth and parenting efforts at the expense reproduction and was relevant even for participants who faced financial unpredictability. During the pandemic, we also observed that mate retention and kin care (family) motives were predominant among individuals who had greater family support during childhood.

**Discussion:**

Overall, the findings suggest that unpredictability experienced during childhood is crucial for the development of life-history strategies and the manifestation of fundamental motives in adulthood.

## 1 Introduction

During the COVID-19 pandemic, the routines of thousands of people were profoundly transformed by changes in hygiene habits and social distancing measures (Broomell et al., 2020; Gao and Li, 2022). During this unique situation, a variation in adherence to preventive measures and the expression of reproductive behaviors was observed. To explain the expression of behaviors like these based on the environmental cues perceived by organisms, we used the life-history (LH) theory. This theory posits that the energy captured by an organism must be allocated across all its physiological processes; however, as the energy supply is finite, trade-offs occur between the energy allocated to somatic effort and reproductive effort, resulting in a fast-slow continuum (Stearns, 1992; Del Giudice and Belsky, 2011; Kruger, 2021). We can observe different LH strategies among species, depending on the ecological context in which they are embedded, or even identify variations in LH trajectories within the same species. For instance, the human species exhibits sufficient phenotypic plasticity to adapt their LH strategy, either making them faster or slower, based on perceived environmental predictability in sensitive periods (Frankenhuis and Fraley, 2017). Among the measures used to assess the LH of our species, there are two fundamental dimensions of environmental variation and influence: harshness and unpredictability (Ellis et al., 2009), and these aspects of the environment influence a diverse range of behaviors in adulthood (Simpson et al., 2012). Thus, the present study aimed to connect LH strategies with the expression of fundamental motives related to self-preservation and the reproductive domain during the pandemic.

### 1.1 Harsh environments

The concept of harsh environments describes external factors capable of causing disability and death for individuals of various ages within a population, and this concept is also addressed by life-history theory (Belsky et al., 2012). Harshness is an environmental condition capable of shaping LH trajectories in response to the environmental conditions experienced throughout development (Ellis, 2004; Chang and Lu, 2017). This is because in harsher environments, with higher mortality rates, having a late start to reproduction may mean not reproducing at all. Therefore, exposure to resource uncertainty and harsh conditions favors the expression of a fast LH trajectory and can be observed through reproductive milestones (Ellis et al., 2009). For example, individuals with a fast LH strategy exhibit earlier sexual debut, early parenthood initiation, a higher number of children by the end of reproductive life, as well as lower parental investment, greater mating effort, and higher risk propensity (Del Giudice et al., 2015). On the other hand, living in more predictable and less harsh environments favors the accumulation of resources for later competition, favoring the strategy of individuals with slow LH trajectories. They achieve reproductive milestones later and display greater parental investment, higher concern for partner retention, and lower risk propensity (Del Giudice et al., 2015). In Western societies, harshness is commonly assessed through socioeconomic status (SES) as lower SES levels are consistently associated with increased rates of morbidity and mortality across various domains (Chen et al., 2002; Ellis et al., 2009; Simpson et al., 2012).

The life-history strategy directly impacts levels of parental investment. This is because one of the expected trade-offs within the LH concerns investing in mating effort or parental effort as an increase in the number of offspring reduces the time and energy available to allocate to each of them (Kruger, 2021). Individuals adopting a fast LH strategy will primarily invest in seeking mates, thereby increasing their reproductive potential; conversely, those following a slow LH strategy will focus their efforts on mate retention and parental care (Del Giudice and Belsky, 2011; Lu et al., 2017). These behavioral expressions align with the theoretical expectations outlined in the parental investment theory (Trivers, 1972), where it is anticipated that men will exhibit more elements of a fast LH when compared to women. This is due to differences in reproductive potential between the sexes, with men increasing their reproductive success by mating with more women, while women increase their reproductive success by being more selective with their mates (Trivers, 1972; Salas-Rodríguez et al., 2021). However, there is an important peculiarity to highlight in the human species: men also contribute to parental care and, therefore, also exhibit aspects of partner selectivity in long-term relationships (Arnocky and Vaillancourt, 2017), resulting in individual differences within each sex. Thus, environmental conditions play a fundamental role in calibrating individuals' LH strategies (Pepper and Nettle, 2017). Therefore, factors such as harshness can lead an individual to adopt a life history different from what is theoretically expected based on sex differences, due to the significant behavioral plasticity found in our species.

### 1.2 Family unpredictability

Unpredictability is also an environmental condition capable of shaping LH trajectories (Ellis, 2004). Following a similar logic, more unpredictable environments signify higher chances of resource scarcity. Therefore, it is evolutionarily advantageous to anticipate reproduction rather than investing more time and energy in maturation or resource accumulation (Lu et al., 2017). Thus, in more stable environments, organisms have a better chance to accumulate resources for competition, favoring the adoption of a slower LH strategy compared to unpredictable contexts, which, in turn, favors faster strategies (Chang et al., 2019a).

The cues of unpredictability that most influence LH trajectories are experienced during childhood (Stearns, 1992; Ellis et al., 2022). The family unpredictability experienced in childhood is the result of inconsistent parental behaviors in meeting the basic needs of children (Howat-Rodrigues and Tokumaru, 2014). According to Hill et al. (2008), this construct derives from attachment theory (e.g., Bowlby, 1969) and life-history theory (e.g., Hill et al., 1997). The mental model of unpredictability is supported by inconsistent family patterns regarding emotional support, financial resources, and mealtime schedules in the early stages of development (Howat-Rodrigues and Tokumaru, 2014). Thus, family unpredictability seems to be the primary cue perceived by the infant, aiding in the calibration of their LH strategy.

During childhood, parenting plays a crucial role. In predictable environments, caregivers have more availability to invest energy and care in their offspring, resulting in higher returns in terms of survival and future reproductive success for their children (Chisholm et al., 1993; Lu et al., 2023). Furthermore, this predictability is transmitted from caregiver to offspring. The opposite occurs in unpredictable environments, where caregivers have less availability for their offspring, resulting in fewer resources and care, contributing to children perceiving greater environmental unpredictability. Therefore, for individuals in their early life stages, the levels of unpredictability experienced in the family environment play a fundamental role in shaping their LH strategies.

Thus, both harshness and family unpredictability contribute to the development of fast LH strategies. This means that, in adulthood, these individuals are inclined to exhibit behaviors consistent with this strategy. For example, these patterns predispose adult individuals to a preference for immediate benefits over future benefits (Hill et al., 2008). Therefore, exposure to inconsistent patterns of family behavior and harsh environments in childhood predicts a higher risk propensity in adulthood (Andrade-Silva et al., 2016), as well as an increased focus on seeking reproductive partners and lower levels of parental care (Neel et al., 2016).

### 1.3 Fundamental social motives

Our ancestors faced a range of recurrent challenges, including the need to prevent diseases, avoid physical harm, gain and maintain status, find mates for reproduction, and care for kin. Kenrick et al. (2010a) developed the concept of fundamental social motives (or simply, fundamental motives), which suggests that humans possess seven distinct motivational systems: self-protection, disease avoidance, affiliation, status pursuit, mate acquisition, mate retention, and parental care (Kenrick et al., 2010a; Neuberg et al., 2011). Each of these systems was shaped over the course of evolution in response to specific challenges encountered by our ancestors, and they are activated by specific environmental cues that lead to physiological and behavioral responses. This theory assumes that in modern times, these fundamental motives still play a central role in our decision-making and social interactions.

The framework of fundamental motives allows for quantifying reproductive and risk-taking behavior. Factors such as age, sex, and exposure to different environmental cues lead to variations in the expression of these motives among individuals (e.g., Kenrick et al., 2010b; Neel et al., 2016). Some of these environmental cues also help explain an individual's LH, as previously mentioned, such as unpredictability and harshness in childhood. Both childhood unpredictability and exposure to harsh environments (Belsky et al., 2012; Simpson et al., 2012) influence the expression of motives related to self-protection, disease avoidance, and reproductive domains in adults (Neel et al., 2016).

Self-protection and disease avoidance are among the first motives to emerge during development. The former is linked to maintaining the individual's physical integrity, while the latter pertains to the behavioral immune system (Neuberg et al., 2011). Although they frequently occur concurrently, both are distinct motivational systems activated by feelings of fear and disgust, respectively (Neel et al., 2016). Based on the previously discussed variables influencing the LH strategy adopted, individuals who experienced greater family unpredictability in childhood (Ross and Hill, 2002) and were exposed to harsh environments (Belsky et al., 2012) tend to exhibit a higher inclination toward risk-taking behaviors in adulthood. Higher levels of childhood unpredictability have also been associated with higher levels of risk-taking in health-related aspects as individuals tend to prioritize other social motives above their long-term health (Martinez et al., 2022). We observe that these indicators, which underlie a fast LH strategy, tend to inhibit the activation of these more fundamental motives associated with an individual's physical integrity and health in adulthood.

As we move toward the end of an individual's development, we have the manifestation of reproductive (mate seeking and mate retention) and caregiving motives. The mate seeking motive is linked to the pursuit of sexual partners, mate retention pertains to maintaining romantic relationships, and parental care relates to caring for children and family (Pick et al., 2022a). Similarly, the variables influencing the LH strategy also impact the expression of motives related to reproduction and caregiving in adulthood. For instance, individuals who have experienced greater family unpredictability and harshness tend to adopt a faster strategy and exhibit higher levels of mate seeking compared to mate retention and parental care (Chang et al., 2019b). Conversely, those who have encountered lower levels of unpredictability and harshness tend to adopt a slower strategy, demonstrating higher levels of mate retention and parental care compared to mate seeking (Chang et al., 2019b). Therefore, throughout an individual's development, the mentioned LH indicators can influence the expression of motives related to the reproductive domain.

### 1.4 Expression of fundamental motives during the pandemic

Despite being primarily transmitted through respiratory means and by asymptomatic individuals, information disseminated through the media has turned coughs and sneezes into stigmas of the disease, even in cases of false positives (Bouayed, 2022). Thus, although the behavioral immune system may not have been as effective in identifying disease signs, given that many individuals were asymptomatic (Ackerman et al., 2021), the study by Keller et al. (2022) demonstrated that watching videos of people sneezing or coughing elicited disgust and fear responses capable of activating the immune system as well, through the production of secretory immunoglobulin A. Therefore, it is expected that witnessing sneezes and coughs generated disgust responses and, thus, activated motives related to disease avoidance. On the other hand, the relationship between self-protection and cues of contagious diseases is indirect. This relationship occurs because the impending threat of contagion can evoke feelings of fear (Galoni et al., 2020), which stem from the establishment of a state of uncertainty and lack of individual control (Lerner and Keltner, 2000, 2001; Galoni et al., 2020). Thus, the pandemic context may have highlighted contagion cues that elicited feelings of disgust and fear, triggering motives of self-protection and disease avoidance.

The fundamental motives related to the reproductive domain were also impacted by the pandemic. The social distancing measures imposed during the pandemic may lead to individuals encountering difficulties in mate seeking as these measures aimed to avoid social contact as a means of reducing the risk of contagion (Pick et al., 2022b). Additionally, confinement led many couples to focus on retaining their partners and caring for their children and/or family members (Ko et al., 2020; Pick et al., 2022b). Therefore, the restrictions imposed by the pandemic affected both individuals who intended to seek partners and those involved in maintaining their relationships and caring for their children.

During the pandemic, there was variation in adherence to preventive measures (Faasse and Newby, 2020), and those who did not follow these guidelines increased their susceptibility to the risk of infection. Risk propensity refers to the tendency to make decisions that may cause physical or psychological harm that threatens survival (Boyer, 2006). In the case of motives related to disease avoidance and self-protection, disregarding interactions with individuals who appear ill can be considered risky behavior. Similarly, continuing to seek partners and disregarding social distancing guidelines can also be seen as risky behavior. Conversely, maintaining one's partner and caring for their offspring may be considered low-risk behavior, especially for cohabiting families. The propensity for risky behaviors varies among individuals and is influenced by LH indicators such as family unpredictability experienced (Ross and Hill, 2002) and exposure to harsh environments (Belsky et al., 2012) in childhood. Thus, it is possible that understanding these LH indicators can increase the explanatory power of why variations were observed in these motivated behaviors among adults during the pandemic.

### 1.5 Present study

In this study, we focus on the expression of fundamental motives related to self-protection, disease avoidance, mate seeking, mate retention, and parental/familial care. It is an exploratory study aiming to investigate whether exposure to different levels of family unpredictability during childhood is associated with reproductive milestones and can explain differences in the expression of motives related to contagion risk and aspects of reproductive behavior during the pandemic. To achieve this, participants were initially grouped based on the similarity of their experiences related to family unpredictability during childhood. Following this procedure, the groups were described and compared in terms of reproductive milestones and the expression of motives related to self-protection, disease avoidance, and reproductive domains. We hypothesize that (a) groups of individuals with different experiences of unpredictability during childhood will exhibit distinct reproductive milestones, and (b) these groups will show differences in the expression of motives related to self-protection, disease avoidance, and reproductive domains.

## 2 Materials and methods

### 2.1 Transparency and openness

This study was not preregistered. All data and analysis code have been made publicly available at the Open Science Framework and can be accessed at https://osf.io/4qvu6/?view_only=b98e70d2d2454b88b688cef016b3896b. The study adhered to the JARS guideline (Kazak, 2018) and the principles outlined in The Code of Ethics of the World Medical Association (Declaration of Helsinki) regarding experiments involving humans.

### 2.2 Sampling procedures

All participants were recruited exclusively through social networks, where the survey was introduced, and a link was provided. The survey was promoted on researchers' personal profiles, university laboratory profiles, and institutional emails. The sample was a convenience sample. The intended sample size (*N* = 252) was calculated using GPower 3.1 software (Mayr et al., 2007) with the following parameters: alpha value of 5%, expected power of 95%, and an effect measure (*f* ) of 0.25. There were 16 participants who failed to fully complete all questionnaires and were thus excluded from the analyses. The achieved sample size was *N* = 438. Data collection took place online using the Google Forms platform from November 2021 to May 2022. All participants volunteered and did not receive any payment. This research was submitted and approved by the local Human Research Ethics Committee with opinion number: 45297521.6.0000.5537. All participants provided their consent for participation and publication of research data through the free and informed consent form (FICF).

### 2.3 Participants

A total of 438 individuals (280 women) over 18 years of age, of both sexes, and residing in Brazil participated in the study. The exclusion criteria were being under 18 years of age, not residing in Brazil at the time of the survey, and not having fully completed all the questionnaires. The mean age of participants was 31.66 years (SD = 10.93). In terms of education, 62.33% reported having completed higher education, 37.44% had completed secondary education, and 0,23% had completed high school. Based on the Brazilian Economic Classification Criteria (Associação Brasileira de Empresas de Pesquisa, 2021), 15.75% of the participants belonged to class A, 56.85% belonged to class B, 24.43% to class C, and 2.97% to class D and E. Class A participants have a higher level of economic power, while individuals from classes D and E have a lower level of economic power.

### 2.4 Measures and instruments

In this research, sociodemographic data, perceived unpredictability in childhood, and levels of activation of fundamental motives were collected. Below, we briefly describe the instruments used.

The *Sociodemographic Measures* consisted of two parts: one focused on sociodemographic characteristics and the other on reproductive milestones in life history. In the first part, questions referred to consumption items and the subject's level of education, which resulted in the categorization of the person's socioeconomic status according to the Brazil Criterion (Associação Brasileira de Empresas de Pesquisa, 2021). The second part contained variables considered relevant in the literature for analyzing the individual's life-history trajectory, such as number of children, age at first sex, age at first child, and menarche.

The *Scale of Family Unpredictability during Childhood*, developed and validated by Howat-Rodrigues and Tokumaru (2014), was used. The questionnaire is divided into three dimensions: Nurturance, Money, and Meals. The components of the Nurturance dimension were associated with parental inconsistency in fulfilling emotional needs and providing help in dealing with challenging circumstances, which we refer to as “family support”. The components comprising the Money factor were associated with uncertainty about the availability of financial resources to cover expenses and purchase goods, which we refer to as “uncertainty of financial resources”. The items included in the Meals factor were associated with uncertainty about mealtime schedules and the individuals who would take part in them, which we refer to as “family mealtime routine”. The scale is a 5-point Likert scale, where 1 corresponds to “strongly disagree” and 5 corresponds to “strongly agree”. The first dimension contained questions such as “I was sure that my family would take care of me”, the second dimension was “In my house, we didn't know if there would be food for daily meals”, and the third dimension was “In my house, I knew who would be present at mealtime”. The internal consistency of the scale measured by Cronbach's alpha was α = 0.858.

The *Fundamental Social Motives Inventory* by Neel et al. (2016) was used to measure motivations related to individual self-protection, disease avoidance, mating effort, and family care. The inventory was translated into Brazilian Portuguese by a group of researchers from Brazil in collaboration with the original instrument researchers. The questionnaire is a 7-point Likert scale, ranging from “strongly disagree” to “strongly agree”. The scales used were self-protection (“I think a lot about how to stay safe from dangerous people”), disease avoidance (“I avoid places and people that might carry diseases”), mate seeking (“I spend a lot of time thinking about ways to meet possible dating partners”), mate retention (“It is important to me that my partner is sexually loyal to me”), kin care—family (“Caring for family members is important to me”), and kin care—children (“I help take care of my children”). Each motive is composed of six questions, and the minimum and maximum possible score is 6 and 36, respectively. The internal consistency of the scale measured by Cronbach's alpha was α = 0.788.

### 2.5 Procedures

The participants were the unit of investigation in this study. Data collection was conducted through an online questionnaire using the Google Forms platform. All participants were recruited via social networks or institutional emails, where a brief introduction to the survey and an access link were provided. Upon reading the FICF and agreeing to participate, participants were directed to a tab containing the instruments to be completed. Once they finished the questionnaire, participants were thanked and dismissed.

### 2.6 Data analysis

We conducted a cluster analysis to group participants based on the similarities in experiences related to family unpredictability during childhood, using the items from the Scale of Family Unpredictability during Childhood (Howat-Rodrigues and Tokumaru, 2014). In this procedure, the ideal number of clusters was determined by grouping participants (cases) according to their questionnaire responses using the method of linkage between groups with squared Euclidean distance. The number of clusters was based on the greatest distance between clustered coefficients in the agglomeration scheme. We conducted a general linear model (GLM) using the clusters to test both of our hypotheses: (a) examining the impact of different patterns of family unpredictability experienced during childhood and sex (independent variables) on reproductive milestones (dependent variables) and (b) investigating the influence of different patterns of family unpredictability experienced during childhood (independent variable) on the participants' scores of the fundamental motives disease avoidance, self-protection, mate seeking, mate retention, kin care (family), and kin care (children) (dependent variables). Statistical significance was set at a level of 5% for all analyses. All data were analyzed using IBM SPSS Statistics, Version 26.

## 3 Results

### 3.1 Clusters

The model that best fits the sample identified three clusters. Cluster 1 consisted of participants with the highest means of family support and family mealtime routine, as well as the lowest uncertainty of financial resources, and was therefore labeled as “low unpredictability”. Both Cluster 2 and Cluster 3 comprised individuals with higher means of uncertainty of financial resources and lower means of family mealtime routine. The distinguishing factor between these two clusters was that Cluster 2 had higher means of family support compared to Cluster 3, resulting in Cluster 2 being labeled as “unpredictability with family support” while Cluster 3 was labeled as “unpredictability without family support”. The means for each cluster are presented in the table below (Table 1).

**Table 1:** Results of ANOVA comparing the ratings of the clusters in terms of the scale of family unpredictability during childhood items.

	**Low unpredictability**	**Unpredictability with family support**	**Unpredictability without family support**
	**M (SD)**	**M (SD)**	**M (SD)**
**Family support**			
I knew my family would be there to take care of me	4.83 (0.44)^a^	4.37 (0.91)^b^	3.18 (1.14)^c^
I was sure that my family would support me if I needed	4.73 (0.51)^a^	4.27 (0.87)^b^	2.77 (1.12)^c^
I felt loved by my family	4.69 (0.59)^a^	4.43 (0.68)^b^	2.51 (1.07)^b^
I knew my family would be there to protect me	4.78 (0.50)^a^	4.41 (0.77)^b^	2.79 (1.15)^c^
I knew I was important for my family	4.70 (0.61)^a^	4.48 (0.68)^b^	2.60 (1.14)^c^
In my family, we cared for each other	4.54 (0.67)^a^	4.13 (0.96)^b^	2.41 (1.11)^c^
When I got my feelings hurt, I went to my mom for comfort	3.85 (1.29)^a^	3.50 (1.33)^b^	1.71 (0.91)^c^
**Uncertainty of financial resources**			
There were times when we did not have money to pay for basic needs (toiletries, clothing, etc.)	1.55 (1.04)^c^	4.26 (0.95)^a^	2.64 (1.45)^b^
My family was never sure whether there would be food for daily meals	1.10 (0.48)^c^	2.91 (1.44)^a^	1.61 (1.04)^b^
When I was a child, my family was never sure how we would pay our bills from month to month	1.51 (0.89)^c^	3.96 (1.07)^a^	2.30 (1.33)^b^
Other children from my family and I had to start working early on	1.26 (0.69)^c^	2.70 (1.64)^a^	1.84 (1.34)^b^
My family was always concerned that we would run out of food before we could buy more	1.36 (0.87)^c^	3.82 (1.13)^a^	2.19 (1.31)^b^
During my childhood, there were people in my family who became unemployed	2.34 (1.53)^c^	4.32 (1.14)^a^	3.00 (1.64)^b^
**Family mealtime routine**			
Usually, dinner was served at the same time everyday	4.31 (1.03)^a^	3.87 (1.40)^b^	3.37 (1.52)^c^
Usually, lunch was served at the same time everyday	4.40 (0.98)^a^	3.91 (1.41)^b^	3.62 (1.38)^b^
During my childhood, the same people sat down and ate dinner Monday through Friday	4.46 (0.92)^a^	3.91 (1.38)^b^	3.28 (1.51)^c^
Meals at my home were at different times everyday	1.64 (1.09)^b^	2.30 (1.54)^a^	2.31 (1.44)^a^
At my home, I knew who would be present at meals	4.46 (0.99)^a^	4.09 (1.27)^b^	3.59 (1.38)^c^

In the comparison between clusters, identical letters indicate equal means for each item according to the *post-hoc* Bonferroni test (*p* < 0.05).

### 3.2 Life-history milestones

Significant differences were observed in the number of children, age at first sex, and age at first reproduction based on clusters, sex, and the interaction between them. It is important to note that the groups did not differ in terms of age. Regarding the number of children, a significant difference was found between clusters, between sexes, and in the interaction between them. In terms of clusters, the “unpredictability without family support” group had a higher mean number of children (M_Unpredic Without Support_ = 2.54, 95% CI: 1.99–3.10, *p* < 0.05) compared to the other two groups (M_Low Unpredictability_ = 1.77, 95% CI: 1.50–2.04; M_Unpredic With Support_ = 1.58, 95% CI: 1.19–1.98). In terms of sex, men had a higher mean number of children (M = 2.24, 95% CI: 1.84–2.65, *p* = 0.026) compared to women (M = 1.69, 95% CI: 1.41–1.96). Regarding the interaction, male individuals in the “unpredictability without family support” group had a higher mean number of children (M = 3.40, 95% CI: 2.45–4.34, *p* < 0.05) compared to all others. The variable “age at first sex” showed a significant difference between clusters and in relation to sex. Among the clusters, the “unpredictability without family support” group had an earlier age at first sex (M_Unpredic Without Support_ = 16.93, 95% CI: 16.14–17.27, *p* =0.007) compared to the “low unpredictability” group (M_Low Unpredictability_ = 18.41, 95% CI: 17.88–18.93), while the “unpredictability with family support” group did not differ from either of the other two groups (M_Unpredic Witht Support_ = 18.03, 95% CI: 17.37–18.69). In terms of sex, men had an earlier age at first sex (M = 17.17, 95% CI: 16.55–17.80, *p* =0.002) compared to women (M = 18.41, 95% CI: 17.95–18.86). The variable “age at first reproduction” showed a difference between sexes, with men having their first child at a later age (M = 29.60, 95% CI: 27.18–32.02, *p* =0.001) compared to women (M = 24.41, 95% CI: 22.73–26.08). No significant differences were found in the variable menarche. The main results described in this subsection can be seen in the table below (Table 2).

**Table 2:** Results of the GLM analysis comparing the clusters, sex, and investigating the effect of the interaction between sex and cluster in terms of reproductive milestones in life history.

	**df**	***F***	***P***	**η^2^ Partial**	**Power**
**Number of children**					
Cluster	2, 103	4.09	**0.019**	0.07	0.71
Sex	1, 103	5.09	**0.026**	0.05	0.61
Cluster ^*^ Sex	2, 103	4.02	**0.021**	0.07	0.71
**Age at first sex**					
Cluster	2, 359	4.70	**0.010**	0.03	0.79
Sex	1, 359	9.89	**0.002**	0.03	0.88
Cluster ^*^ Sex	2, 359	1.30	0.273	0.01	0.28
**Age at first reproduction**					
Cluster	2, 104	1.45	0.239	0.03	0.30
Sex	1, 104	12.22	**0.001**	0.11	0.93
Cluster ^*^ Sex	2, 104	1.67	0.192	0.03	0.35
**Menarche**					
Cluster	2, 263	2.23	0.109	0.02	0.45

In bold, *p* < 0.05.

### 3.3 Fundamental motives

The GLM tests showed differences among the clusters in relation to the fundamental domains of “mate retention” and “kin care (family)” (Table 3). Bonferroni's *post-hoc* test revealed that the “unpredictability without family support” group exhibited fewer scores related to both “mate retention” and “kin care (family)” motivations. The “low unpredictability” group and the “unpredictability with family support” group did not differ from each other in terms of both motivations.

**Table 3:** Results of the GLM analysis comparing the clusters in terms of the fundamental motives self-protection, disease avoidance, mate seeking, mate retention, kin care (family), and kin care (children).

	**M (95% CI)**	**df**	***F***	***p***	**η^2^ partial**	**Power**
**Self-protection**						
Low unpredictability	32.51 (31.42–33.61)^a^					
Unpredictability with family support	32.70 (31.29–34.11)^a^	2, 435	1.71	0.181	0.008	0.360
Unpredictability without family support	30.80 (29.10–32.50)^a^					
**Disease avoidance**						
Low unpredictability	29.93 (28.85–31.02)^a^					
Unpredictability with family support	29.62 (28.22–31.02)^a^	2, 435	0.06	0.940	< 0.001	0.059
Unpredictability without family support	29.88 (28.20–31.57)^a^					
**Mate seeking**						
Low unpredictability	16.26 (14.90–17.63)^a^					
Unpredictability with family support	17.35 (15.59–19.11)^a^	2, 435	1.81	0.165	0.008	0.378
Unpredictability without family support	18.66 (16.54–20.78)^a^					
**Mate retention**						
Low unpredictability	35.99 (34.95–37.03)^a^					
Unpredictability with family support	34.25 (32.85–35.66)^ab^	2, 298	7.48	**0.001**	0.048	0.941
Unpredictability without family support	31.90 (30.02–33.77)^b^					
**Kin care (family)**						
Low unpredictability	37.06 (36.14–37.98)^a^					
Unpredictability with family support	35.30 (34.12–36.49)^a^	2, 435	52.92	**< 0.001**	0.196	1.000
Unpredictability without family support	28.22 (26.79–29.65)^b^					
**Kin care (children)**						
Low unpredictability	37.09 (35.69–38.49)^a^					
Unpredictability with family support	36.24 (34.37–38.17)^a^	2, 111	1.39	0.254	0.024	0.293
Unpredictability without family support	34.75 (32.30–37.19)^a^					

In the comparison between clusters, identical letters indicate equal means for each item according to the *post-hoc* Bonferroni test (*p* < 0.05) and in bold, *p* < 0.05.

## 4 Discussion

The study aimed to investigate whether different experiences of unpredictability in childhood influence the occurrence of reproductive milestones and help explain the expression of fundamental motives in adult individuals during the COVID-19 pandemic.

We observed that, in general, individuals with higher childhood unpredictability have more children and an earlier onset of sexual activity. This pattern suggests that unpredictability supports a fast life-history strategy (Ellis et al., 2022). Family unpredictability affects development, and higher levels of family support indicate more time, attention, and parental investment directed toward children (Simpson et al., 2012). Several studies have found that the quality of parental care can mediate the relationship between childhood adversities and behaviors exhibited in adolescence and adulthood (Belsky et al., 2012; Szepsenwol et al., 2015, 2017, 2019). Specifically, we observed that unpredictability in family support had a significant impact on the expression of these milestones. Among those who experienced financial unpredictability, individuals who had family support showed a similar number of children and onset of sexual activity compared to those who experienced low unpredictability. Although some studies have found associations between low socioeconomic levels in childhood and the display of behavioral traits linked to a fast strategy (Griskevicius et al., 2011a,b, 2013), our findings are consistent with studies indicating that family support in childhood can attenuate the effects of income on reproductive strategy (Szepsenwol et al., 2015, 2017).

Childhood unpredictability influenced men differently as those who experienced unpredictability in childhood without family support have more children than women and men in the other groups. This result is related to the primary trade-off expected for men, which is the difference between mating effort and parental effort (Del Giudice and Belsky, 2011). In a fast life-history strategy, it is expected that mating effort is greater than parental effort, resulting in a higher number of offspring (Geary, 2015). Our finding suggests that low levels of family support have a more intense impact on the expression of reproductive milestones in men. This effect is more pronounced in men than in women, due to the greater variation in reproductive potential typically found in men (Archer, 2009; Zhu and Chang, 2019). This finding also supports the importance of family support in perceiving environmental cues that predict reproductive strategy.

Compared to women, men have more children, start their sexual life earlier, and have children later in life. Additionally, no differences were found in the expression of reproductive milestones regarding unpredictability among women, highlighting that the observed sex differences can be explained by the asymmetry in parental investment between men and women (Trivers, 1972). This occurs because, in mammals, male reproduction has fewer physiological and temporal constraints compared to female reproduction, allowing them to have a greater capacity to generate offspring within the same timeframe, even when both sexes adopt a fast life-history strategy (Geary, 2015). The same logic applies to the early initiation of sexual life. In contexts of unpredictability, men tend to have lower reproductive costs in a fast strategy, which can make it advantageous to start their sexual life earlier compared to women, who face fundamental costs such as pregnancy and lactation. Finally, the later age at which men have children may be related to a specific characteristic of our sample: higher educational and socioeconomic levels. In Western and industrialized societies, the pursuit of higher levels of education is associated with careers that result in higher status and, consequently, higher salaries (Lent and Brown, 2013). Therefore, delayed fatherhood may result from the fact that many men accumulate resources to acquire status and become attractive partners, which involves dedicating more years to their studies. On the other hand, women have higher fertility and reproductive value in early adulthood (Lassek and Gaulin, 2019), which may explain why they have children earlier.

In our sample, no effects of childhood unpredictability on age at menarche were found. Studies addressing this topic have yielded mixed results (Webster et al., 2014), suggesting that father's absence, low financial resources, and a harsh family environment may be associated with earlier menarche (Yermachenko and Dvornyk, 2014). However, the effects of financial scarcity and a harsh family environment were not identified in our results, while the variable of the father's absence was not assessed in this study.

We also investigated the effect of childhood unpredictability on the expression of motives related to self-protection and reproduction. The results indicate that the groups show similar values for the motives of “self-protection” and “disease avoidance”. Childhood unpredictability increases exposure to risky situations (Del Giudice, 2009; Ellis et al., 2009); thus, one would expect lower activation of “self-protection” and “disease avoidance” motives in individuals who experienced high unpredictability as lower activation of these motives would result in greater exposure to the risk of contagion during the pandemic. However, contrary to our expectations, the results indicated that individuals who experienced both high and low unpredictability in childhood showed similar and high scores for the “self-protection” and “disease avoidance” motives. This finding suggests that the threat experienced during the pandemic may have been the predominant stimulus for activating these motivations, overriding the effect of childhood unpredictability.

Among the reproductive motives, we found that individuals without family support in childhood showed lower expression of the “mate retention” and “kin care (family)” motives. As discussed earlier, childhood unpredictability is associated with faster life trajectories and, consequently, lower expression of behaviors focused on partner retention and parental care in adulthood (Belsky et al., 2012; de Baca and Ellis, 2017). The findings indicate that family support in childhood supports the expression of motives associated with a slower strategy. It is interesting to note that both motivations are related to maintaining family bonds, whether they are blood relatives (family) or not (romantic partners) (Kenrick and Lundberg-Kenrick, 2022). This suggests, once again, that even for individuals who experienced other types of unpredictability (such as financial and dietary routines), family support was the most relevant factor in the expression of parental investment (Szepsenwol et al., 2015, 2017).

During the pandemic, participants from all groups showed low scores for “mate seeking” and high scores for “kin care (children)”. According to the literature (Belsky et al., 2012; de Baca and Ellis, 2017), it was expected that childhood unpredictability would be associated with higher expression of the “mate seeking” motive and lower expression of “kin care (children)”. The lack of differences between the groups indicates that, although childhood unpredictability is associated with a faster life trajectory, our sample did not exhibit evidence of these patterns during the pandemic. Regarding “mate seeking”, a possible explanation is that the social restrictions imposed on individuals may have desensitized the expression of this motive. Higher socioeconomic levels have been associated with greater adherence to social distancing behaviors (Broomell et al., 2020), and a significant portion of our sample consists of individuals from such conditions. Therefore, adherence to movement restrictions during the pandemic may have led to a decrease in behaviors focused on seeking partners, overriding individual life trajectories. Finally, the high score for the “kin care (children)” motivation may have originated from feelings of insecurity and the difficulties faced during the pandemic, resulting in efforts directed toward investing time and care in offspring. Such efforts are directly associated with individuals' fitness, occurring regardless of their life trajectory.

The present study has limitations. First, due to the pandemic, data collection was conducted online, resulting in a predominantly middle-class sample with a high level of education (completed high school/completed higher education). For future research, it would be interesting to expand data collection to individuals with lower socioeconomic status and lower levels of education to achieve a greater representation of the Brazilian population. Another limitation, also stemming from online data collection, relates to the use of self-report measures. Future research could explore this topic by incorporating, for example, implicit measures to replicate the study. Finally, our data suggest that the pandemic context may have been responsible for the observed effects; however, the exploratory study design does not allow us to infer this level of causality. It is proposed that future experimental studies corroborate this potential effect.

In this study, we found that childhood unpredictability influences how reproductive milestones are manifested and how reproductive and self-protection motives are expressed. We observed that the family support experienced during childhood predicts a slow life-history strategy, which is relevant even for participants who faced financial uncertainty. During the pandemic, we also observed that mate retention and kin care (family) motives were predominant in individuals who experienced greater family support during childhood. Overall, the findings suggest that childhood unpredictability is crucial for the development of LH strategies and the expression of fundamental motives in adulthood. In conclusion, our findings highlight the importance of family support in childhood for reproductive milestones and the expression of fundamental motives. Additionally, our data suggest that the pandemic acted as desensitizing factors (in terms of risk behaviors) or activators (in relation to care for offspring) of fundamental motives.

## Data availability statement

The original contributions presented in the study are included in the article/supplementary material, further inquiries can be directed to the corresponding author.

## Ethics statement

The studies involving humans were approved by Human Research Ethics Committee - UFRN - with opinion number: 45297521.6.0000.5537. The studies were conducted in accordance with the local legislation and institutional requirements. The participants provided their written informed consent to participate in this study.

## Author contributions

NT: Conceptualization, Investigation, Methodology, Project administration, Resources, Validation, Visualization, Writing – original draft, Writing – review & editing. FC: Formal analysis, Resources, Supervision, Validation, Visualization, Writing – original draft, Writing – review & editing.
